# Supplementary material for: Patterns of Intron Gain and Loss in Fungi
Source: PLoS Biol. 2004 Nov 30;2(12):e422. doi: 10.1371/journal.pbio.0020422 (PMC532390; doi:10.1371/journal.pbio.0020422)
Supplement: Table S1 — Also available at http://genes.mit.edu/NielsenEtAl/. (4.3 MB ZIP). [file pbio.0020422.st001.zip › NielsenEtAl/html/94.html]

AN2302.1.NCU08991.1.MG04978.1.FG06922.1


```
 CLUSTAL W (1.82) Multiple Sequence Alignments - Introns Inserted


Sequence 1: AN2302.1	160 aa
Sequence 2: FG06922.1	169 aa
Sequence 3: NCU08991.1	171 aa
Sequence 4: MG04978.1	168 aa
Alignment Length: 171 aa
Number Identitical Residues: 108 aa
Alignment Score (without introns) 4854


MG04978.1 	MSEGQ---LQKVNLQSNDGQSIEVD1RAVACRSRLIKDLIGDLGEEMVASTPIPIPN~VS
NCU08991.1	MAENDERALQKVSLQSNDGQIITVD1RVVAERSLLIKNLIEDLGDEAVMNEAIPLPN~VN
FG06922.1 	MSEST--SPQKVWLASNDSANIEVD1RVVAERSMLIKNMLEDVGDEITQGNPIPIPN0VN
AN2302.1  	MAE------KMLVMTSNDGKNIEVP1RDVAERSLLIKNMLEDLGDPT---EPIPIPN0VS
          	*:*      : : : ***.  * *  * ** ** ***::: *:*:      .**:** *.

MG04978.1 	EAVLRKVLEWCEHHRNDPVQTSDEDSESRKKTTDIDEWDQKFMQVDQEMLFEIIL0ASNY
NCU08991.1	EPVLRKVVEWCEHHRKDPPQTTEDENDSRKKSTEIDEWDQKFMQVDQEMLFEIIL0AANY
FG06922.1 	EAVLRKVIEWCDHHRNDPPQAQDDESDARKKTTDIEEWDQKFMQVDQEMLFEIIL0ASNY
AN2302.1  	ENVLSKVLEWCAHHRNDPPSSADDD-DSRRKTTDIEEWDQKFMQVDQEMLFEIIL0AANY
          	* ** **:*** ***:** .: ::: ::*:*:*:*:******************* *:**

MG04978.1 	LDIKPLLDVGCKTVANMIKGKSPEEIRKTFNITNDFTPEEEEQIRRENEWAEDR
NCU08991.1	MDIKPLLDVGCKTVANMIKGKSPEEIRKTFNITNDFTPEEEEQIRRENEWAEDR
FG06922.1 	LDIKPLLDVGCKTVANMIKGKAPEEIRKTFNITNDFTPEEEEQIRRENEWAEDR
AN2302.1  	LDIKPLLDIGCKTVANMIKGKSPEEIRKTFNIQNDFTPEEEDQIRRENEWAEE-
          	:*******:************:********** ********:**********:
```
